# Supplementary material for: Catching some air: a method to spatially quantify aerial triazole resistance in Aspergillus fumigatus
Source: Appl Environ Microbiol. 2024 Jun 6;90(7):e00271-24. doi: 10.1128/aem.00271-24 (PMC11267943; doi:10.1128/aem.00271-24)
Supplement: Effect of seals on triazole selectivity — Description of a supplementary experiment where we tested for an effect of the seals on the selectivity of triazoles on growth of A. fumigatus. [file aem.00271-24-s0005.docx]

**Supplement - Effect of the sticky seals on triazole selectivity**

Here we tested to see whether culturing exposed seals by adding the medium with different triazole concentrations directly on top would affect the selectivity of Itraconazole, voriconazole and tebuconazole for triazole-resistant *Aspergillus fumigatus* colonies. We used plating of spore suspensions on the agar surface with glass beads as a positive control treatment for selectivity by the triazoles. For this experiment we cut the sticky seals into small fragments (4 cm x 6,85 cm) and placed them sticky-side-up inside 9 cm Petri dishes. *A. fumigatus* strains with MIC values ranging from 0.5 to 16 mg/L (see Table 1) were included in this experiment. We prepared spore suspensions with a concentration of 1 to 100 spores per 100 µl. We then pipetted 100 µl of spore suspension in 15 to 20 randomly placed separate droplets across the seal (see Figure 1), such that colonies would not be clustered. The droplets were left to air-dry for an hour under a laminar flow hood. Subsequently, we poured 8 ml of 60 °C Flamingo medium over the seals and left to dry at room temperature. For the surface plating control treatment, the plates were poured first and left to dry, then 100 µl of spore suspension was spread over the agar surface with sterile glass beads. All the plates were incubated for three days at 48 °C. Plates were scored qualitatively for the growth of CFUs hereafter. The triazole concentrations included in the experiment are given in Table 1.


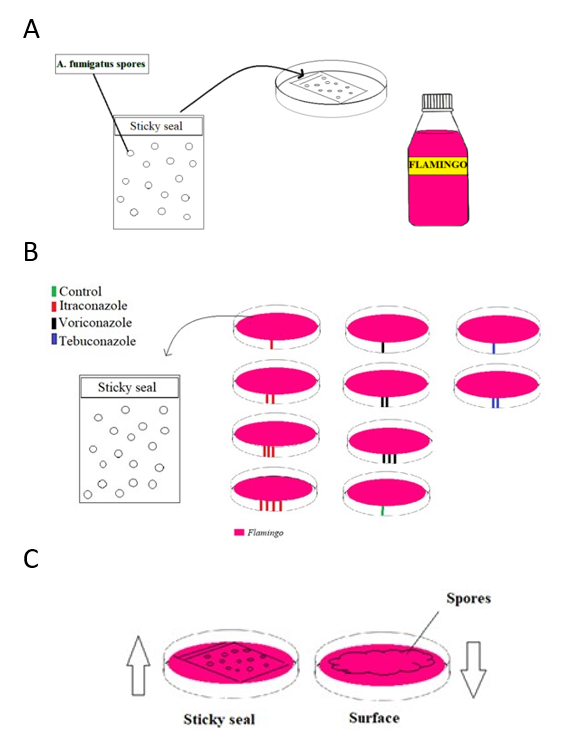


Figure 1: Schematic representation of experimental set-up. A) Seals were placed on the bottom of the Petri dish with droplets of spore suspension placed on them, air dried, and subsequently covered with flamingo medium. B) The increasing number of stripes per colour marks the increasing triazole concentrations in the plates and corresponds with concentrations shown in Table 1. C) As a positive control treatment for triazole selectivity A. fumigatus spore suspensions of the selected strains were also inoculated on the surface of the agar.

Table 1: **Qualitative scores of growth on Flamingo medium with triazole treatment directly on sticky seals.** Triazole treatments included no triazole (0), itraconazole (ITR), voriconazole (VOR) and tebuconazole (TEB). Numbers indicate the concentration of the respective triazole in the agar in mg/L. MIC values of the strains to Itraconazole and voriconazole are given in mg/L.

| **Strain** | **TR type** | **MIC ITR** | **MIC VOR** | **0** | **ITR 4** | **ITR 6** | **ITR 8** | **ITR 10** | **VOR 4** | **VOR 5** | **VOR 6** | **TEB 4** | **TEB 6** |
| --- | --- | --- | --- | --- | --- | --- | --- | --- | --- | --- | --- | --- | --- |
| **AfIR974** | **WT** | **0,5** | **1** | **+**  **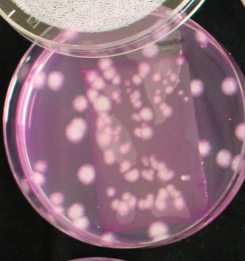** | **+**  **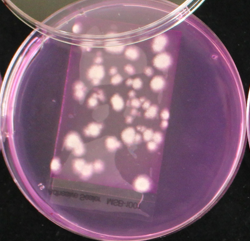** | **+**  **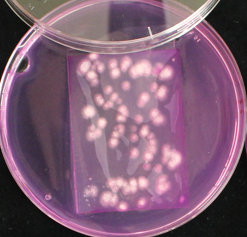** | **+**  **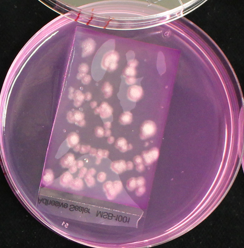** | **+**  **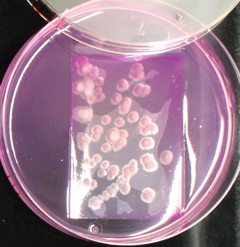** | **-**  **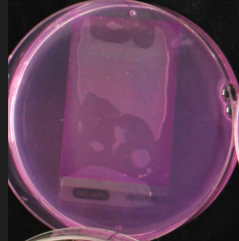** | **-**  **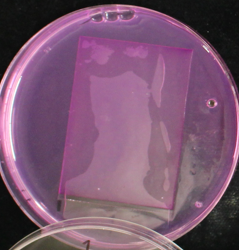** | **-**  **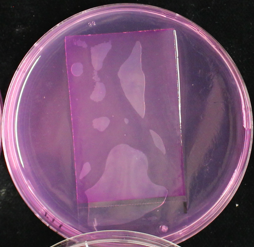** | **+**  **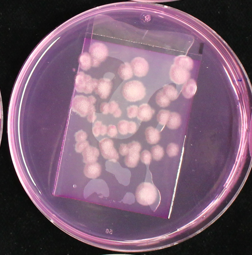** | **+**  **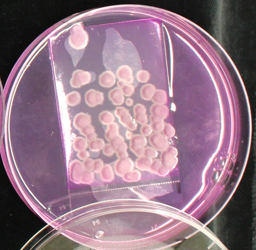** |
| **AfIR964** | **WT** | **0,5** | **1** | **+**  **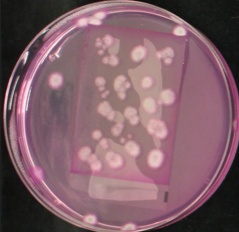** | **+**  **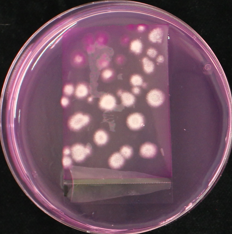** | **+**  **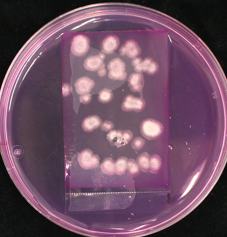** | **+**  **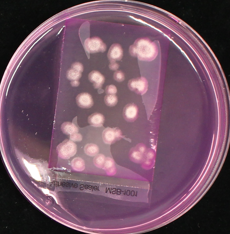** | **+**  **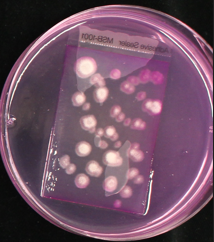** | **-**  **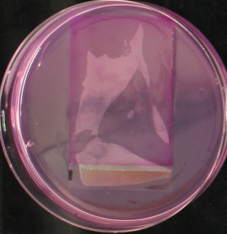** | **-**  **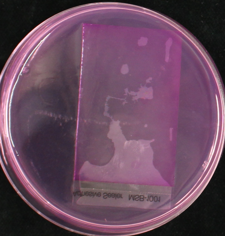** | **-**  **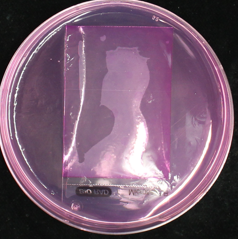** | **+**  **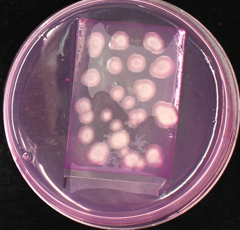** | **+**  **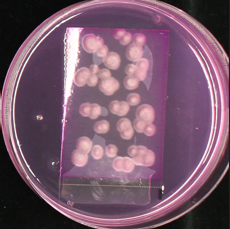** |
| **78-2** | **TR34/L98H** | **16** | **4** | **+**  **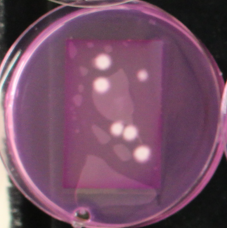** | **+**  **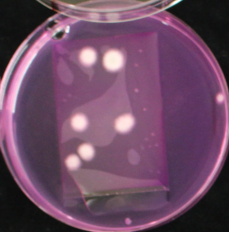** | **+**  **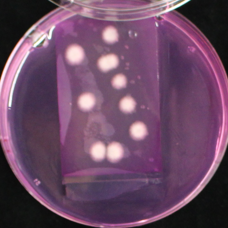** | **+**  **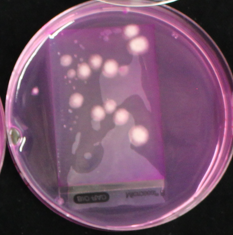** | **+**  **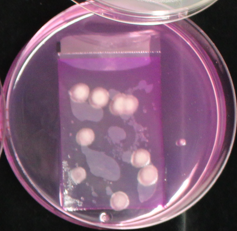** | **+**  **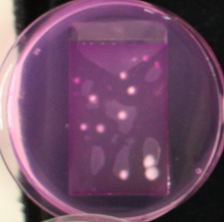** | **+**  **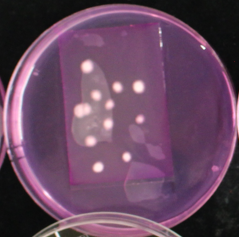** | **-/+**  **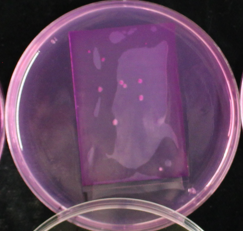** | **+**  **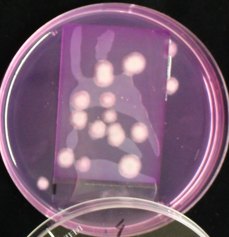** | **+**  **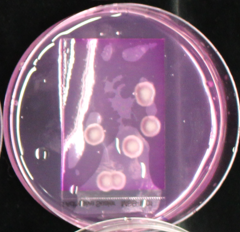** |
| **39-3** | **TR34/L98H /S297T** | **16** | **1** | **+**  **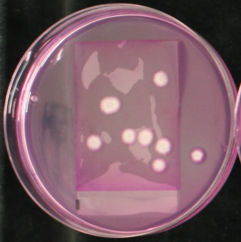** | **+**  **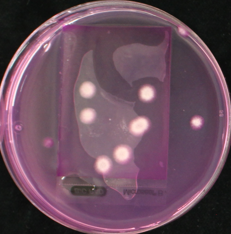** | **+**  **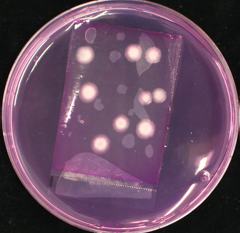** | **+**  **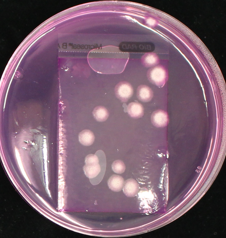** | **+**  **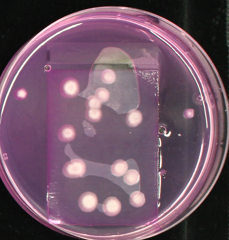** | **-**  **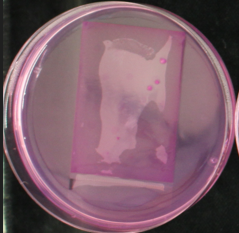** | **-**  **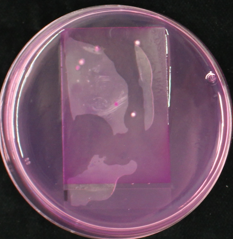** | **-**  **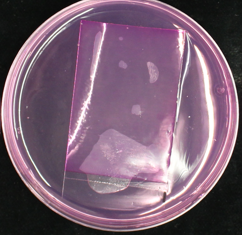** | **+**  **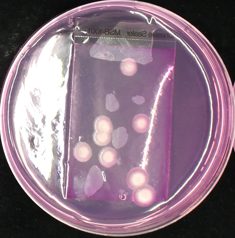** | **+**  **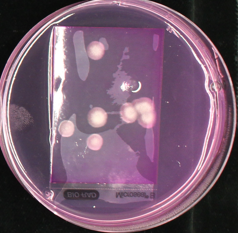** |
| **19-2** | **TR46/Y121F/T289A** | **1** | **16** | **+**  **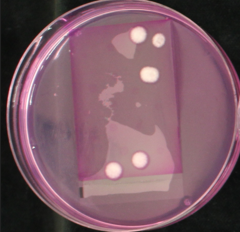** | **+**  **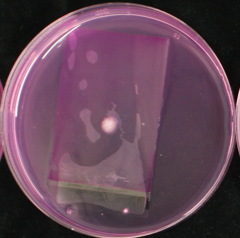** | **+**  **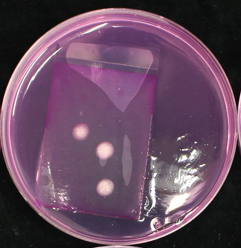** | **+**  **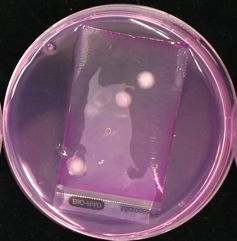** | **+**  **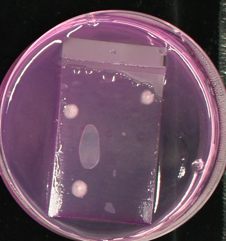** | **+**  **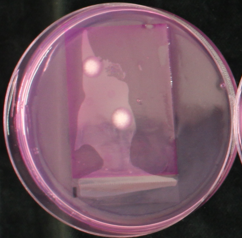** | **+**  **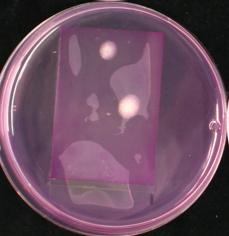** | **+**  **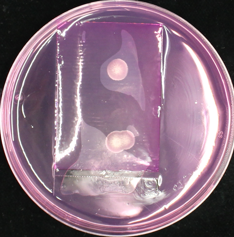** | **+**  **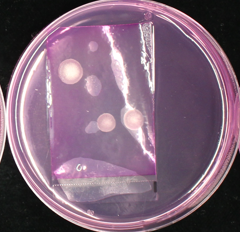** | **+**  **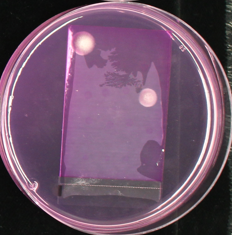** |
| **40-1** | **TR46/Y121F/T289A /S363P /I364V /G448S** | **2** | **16** | **+**  **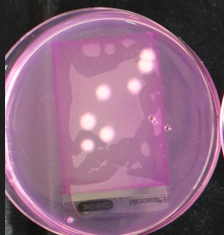** | **+**  **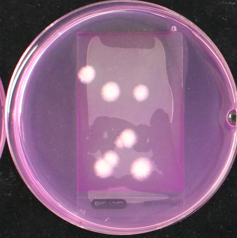** | **+**  **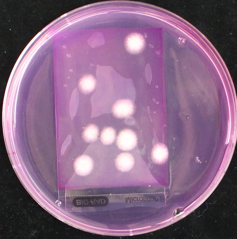** | **+**  **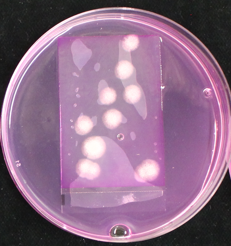** | **+**  **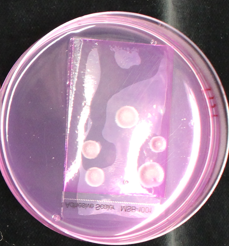** | **+**  **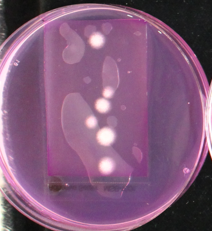** | **+**  **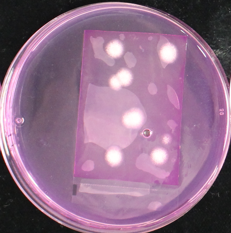** | **+**  **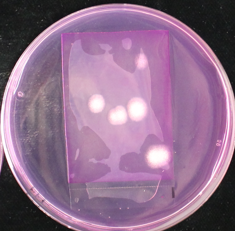** | **+**  **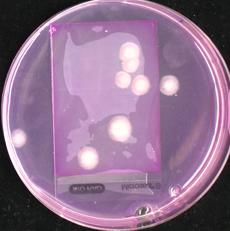** | **+**  **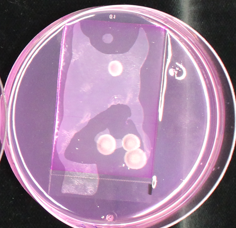** |

Table 2: **Qualitative scores of growth on Flamingo medium with triazole treatment agar surface plating.** Triazole treatments included no triazole (0), itraconazole (ITR), voriconazole (VOR) and tebuconazole (TEB). Numbers indicate the concentration of the respective triazole in the agar in mg/L. MIC values of the strains to Itraconazole and voriconazole are given in mg/L.

| **Strain** | **TR type** | **MIC ITR** | **MIC VOR** | **0** | **ITR 4** | **ITR 6** | **ITR 8** | **ITR 10** | **VOR 4** | **VOR 5** | **VOR 6** |
| --- | --- | --- | --- | --- | --- | --- | --- | --- | --- | --- | --- |
| **AfIR974** | **WT** | **0,5** | **1** | **+** | **-** | **-** | **-** | **-** | **-** | **-** | **-** |
| **AfIR964** | **WT** | **0,5** | **1** | **+** | **-** | **-** | **-** | **-** | **-** | **-** | **-** |
| **78-2** | **TR34/L98H** | **16** | **4** | **+** | **+** | **+** | **+** | **+** | **-** | **-** | **-** |
| **39-3** | **TR34/L98H /S297T** | **16** | **1** | **+** | **+** | **+** | **+** | **+** | **-** | **-** | **-** |
| **19-2** | **TR46 /Y121F /T289A** | **1** | **16** | **+** | **-** | **-** | **-** | **-** | **+** | **+** | **+** |
| **40-1** | **TR46 /Y121F /T289A /S363P /I364V /G448S** | **2** | **16** | **+** | **+** | **+** | **-** | **-** | **+** | **+** | **+** |

**Conclusion**

While growth of the strains corresponded with their MICs when plated on the agar surface (see Table 2), with low-level itraconazole resistance of strain 40-1 being the one exception, we found that there were strong discrepancies when the strains were grown from the seals through the medium (see Table 1). There was growth on all itraconazole plates across all concentrations and reference strains. There was little to no visible reduction of growth even at 10 mg/L, regardless of the MIC value of the strain. For Voriconazole, this effect was much weaker, with only strain 78-2 showing slight growth at concentrations on or above its intermediate voriconazole MIC of 4 mg/L. We conclude that, when applying the Flamingo medium with itraconazole directly on top of the seal, this culturing approach is unreliable for determining itraconazole resistance fractions. For tebuconazole, we also observed growth on all sticky seal plates regardless of strain or applied concentration, but we did not include reference strains with known tebuconazole MIC values or agar surface plating controls for Tebuconazole in this experiment.
